# Clinical efficacy of different monoclonal antibody regimens among non-hospitalised patients with mild to moderate COVID-19 at high risk for disease progression: a prospective cohort study

**DOI:** 10.1007/s10096-022-04464-x

**Published:** 2022-06-21

**Authors:** Alessia Savoldi, Matteo Morra, Pasquale De Nardo, Anna Maria Cattelan, Massimo Mirandola, Vinicio Manfrin, Piergiorgio Scotton, Maria Teresa Giordani, Lucio Brollo, Sandro Panese, Massimiliano Lanzafame, Giovanna Scroccaro, Matilda Berkell, Giuseppe Lippi, Angelina Konnova, Mathias Smet, Surbhi Malhotra-Kumar, Samir Kumar-Singh, Evelina Tacconelli, the mAb Working Group, Marco Canova, Fabio Rigo, Davide Coletto, Francesco Saverio Serino, Ilaria Coledan, Elisa Danese, Denise Peserico, Matteo Gelati, Michela Conti, Daniele Fasan, Basil Britto Xavier, Akshita Gupta, An Hotterbeekx, Paola De Ambrosis

**Affiliations:** 1 Division of Infectious Diseases, Department of Diagnostics and Public Health, University of Verona, P.le L.A. Scuro 10, 37134 Verona, Italy; 2 Infectious Disease Unit, Hospital of Padua, Via Giustiniani 2, 35128 Padua, Italy; 3 School of Health Sciences, University of Brighton, Brighton, UK; 4 Division of Infectious and Tropical Diseases, S. Bortolo Hospital, Viale Ferdinando Rodolfi 37, 36100 Vicenza, Italy; 5 Infectious Diseases Unit, Azienda ULSS2 Marca Trevigiana, Treviso, Italy; 6 Infectious Diseases Unit, Alto Vicentino Santorso Hospital, Azienda ULSS 7via Garziere 42, Santorso, Vicenza, Italy; 7 Division of Internal Medicine and Cardiology, Infectious Diseases and COVID-19 Section, Jesolo Hospital Via Levantina, 104, 30016 Jesolo, Italy; 8 Infectious Diseases Unit, Azienda ULSS 3 Serenissima, Ss. Giovanni E Paolo Hospital, Castello 6777, 30122 Venice, Italy; 9 Division of Infectious Diseases, Ospedale Santa Maria Della Misericordia Hospital, Viale Tre Martiri 140, Rovigo, Rovigo Italy; 10 Department of Pharmaceutical and Devices, Venice, Veneto Region Italy; 11 Lab of Medical Microbiology, Vaccine & Infectious Disease Institute, University of Antwerp, Antwerp, Belgium; 12 Molecular Pathology Group, Cell Biology & Histology, Faculty of Medicine and Health Sciences, University of Antwerp, Antwerp, Belgium; 13 Section of Clinical Biochemistry, University of Verona, Verona, Italy

**Keywords:** Monoclonal antibody treatments for COVID-19, Mild-to-moderate COVID-19 outpatients, Bamlanivimab-etesevimab, casirivimab-imdevimab, SARS-CoV-2 early treatments

## Abstract

This study aimed to compare the clinical progression of COVID-19 in high-risk outpatients treated with the monoclonal antibodies (mAb) bamlanivimab, bamlanivimab-etesevimab and casirivimab-imdevimab. This is an observational, multi-centre, prospective study conducted from 18 March to 15 July 2021 in eight Italian tertiary-care hospitals including mild-to-moderate COVID-19 outpatients receiving bamlanivimab (700 mg), bamlanivimab-etesevimab (700–1400 mg) or casirivimab-imdevimab (1200–1200 mg). All patients were at high risk of COVID-19 progression according to Italian Medicines Agency definitions. In a patient subgroup, SARS-CoV-2 variant and anti-SARS-CoV-2 serology were analysed at baseline. Factors associated with 28-day all-cause hospitalisation were identified using multivariable multilevel logistic regression (MMLR) and summarised with adjusted odds ratio (aOR) and 95% confidence interval (CI). A total of 635 outpatients received mAb: 161 (25.4%) bamlanivimab, 396 (62.4%) bamlanivimab-etesevimab and 78 (12.2%) casirivimab-imdevimab. Ninety-five (15%) patients received full or partial SARS-CoV-2 vaccination. The B.1.1.7 (Alpha) variant was detected in 99% of patients. Baseline serology showed no significant differences among the three mAb regimen groups. Twenty-eight-day all-cause hospitalisation was 11.3%, with a significantly higher proportion (*p* 0.001) in the bamlanivimab group (18.6%), compared to the bamlanivimab-etesevimab (10.1%) and casirivimab-imdevimab (2.6%) groups. On MMLR, aORs for 28-day all-cause hospitalisation were significantly lower in patients receiving bamlanivimab-etesevimab (aOR 0.51, 95% CI 0.30–0.88 *p* 0.015) and casirivimab-imdevimab (aOR 0.14, 95% CI 0.03–0.61, *p* 0.009) compared to those receiving bamlanivimab. No patients with a history of vaccination were hospitalised. The study suggests differences in clinical outcomes among the first available mAb regimens for treating high-risk COVID-19 outpatients. Randomised trials are needed to compare efficacy of mAb combination regimens in high-risk populations and according to circulating variants.

## Introduction

Neutralising monoclonal antibodies (mAb), developed from convalescent COVID-19 patients, target the surface of SARS-COV-2 spike glycoprotein that mediates the viral entry into host cells and represent a promising treatment option for early-stage COVID-19 [1]. In March 2021, the Italian Medicines Agency (AIFA) issued the emergency use approval (EUA) for three neutralising SARS-CoV-2 spike-protein mAb—bamlanivimab, bamlanivimab-etesevimab and casirivimab-imdevimab—for the treatment of mild-to-moderate COVID-19 in paediatric and adult patients at high risk for disease progression [2–4]. The mAb EUAs were based on data from early clinical randomised controlled trials (RCT), demonstrating a decrease in viral load, hospitalisation rate and emergency department (ED) visits in patients receiving mAb compared with placebo, especially when they were administered early after symptom onset [5–7]. While the findings of the placebo-controlled trials are similar for bamlanivimab, bamlanivimab-etesevimab and casirivimab-imdevimab, no studies have yet examined the comparative clinical efficacy among these three mAb regimens.

This study aimed to assess and compare clinical progression of mild-to-moderate COVID-19 in three cohorts of patients at high risk for disease progression who received therapy with bamlanivimab, bamlanivimab-etesevimab or casirivimab-imdevimab in order to identify differences in outcomes among the three mAb regimens.

## Material and methods

### Study design and setting

This is a multi-centre observational prospective study of patients receiving mAb therapy in eight tertiary-care hospitals located in the Veneto Region (Italy). The study protocol was approved by the local ethics committees of each involved centre, and written informed consent was gathered for each patient participating in the study. All procedures were in accordance with the 1964 Helsinki declaration and its later amendments or comparable ethical standards.

### Study population and eligibility criteria

An ad hoc electronic reporting system was developed by the Veneto Region to facilitate the identification of eligible outpatients by general practitioners or ED physicians and hospital centres responsible for mAb administration. From 18 March to 15 June 2021, all signalled patients aged ≥ 12 years with a microbiologically documented SARS-CoV-2 infection (either by polymerase chain reaction or III generation antigenic test on nasopharyngeal swab (NPS)), presenting mild-to-moderate COVID-19 symptoms ≤ 10 days, deemed at high risk for disease progression were offered mAb therapy. Patients ≥ 18 years who signed the informed consent were included in the study. According to AIFA EUA indications, patients were considered eligible for mAb administration if they presented at least one of the medical conditions listed in Table 1. Mild-to-moderate COVID-19 was defined by scores 2 (symptomatic, independent) or 3 (symptomatic, assistance needed) of the World Health Organization (WHO) Clinical Progression Score [8]. Outpatients received a one-time fixed-dose intravenous infusion of bamlanivimab 700 mg, or bamlanivimab-etesevimab 700–1400 mg, or casirivimab-imdevimab 1200–1200 mg. The decision on the type of mAb to be administered was based on the available supply at various hospitals. Follow-up continued until 15 July 2021 (completion of 28-day follow-up of the last treated patient).

**Table 1:** Italian Medicines Agency emergency use authorisation eligibility criteria (high-risk patients for COVID-19 clinical progression) for bamlanivimab, bamlanivimab-etesevimab and casirivimab-imdevimab therapy in adult patients

All the following criteria should be met:
1) Confirmed diagnosis of SARS-CoV-2 infection either by polymerase chain reaction or III generation antigenic test on nasopharyngeal swab
2) Onset of at least one of the COVID-19 related symptoms among fever, cough, dyspnoea, headache, myalgia, gastro-intestinal symptoms, asthenia ≤ 10 days
3) Age ≥ 12 years
4) Body weight ≥ 40 kg
5) No need for oxygen therapy
6) No need for hospitalisation
7) Presence of at least one of the following medical conditions:
a. BMI ≥ 35 kg/m^2^
b. Subject chronically undergoing peritoneal dialysis or haemodialysis
c. Uncontrolled diabetes mellitus (HbA1c ≥ 9% or 75 mmol/L) or with chronic complications
d. Primary immunodeficiency
e. Secondary immunodeficiency (e.g. hematologic cancer patient in ongoing myelo/immunosuppressive therapy or suspension for < 6 months)
f. Cardio-cerebrovascular disease (including arterial hypertension with documented organ damage) in subjects aged ≥ 55 years
g. Chronic obstructive pulmonary disease and/or other chronic respiratory disease in subjects ≥ 55 years

### Variable description and data collection

The following variables were collected for each patient at the time of study inclusion: age, gender, time from COVID-19 symptoms onset to mAb infusion (days), type of medical conditions which qualified the patient for mAb therapy, namely: (i) body mass index (BMI) ≥ 35 kg/m^2^, (ii) chronic dialysis, (iii) any cardio-cerebrovascular disease in subject ≥ 55 years (including arterial hypertension with documented organ damage), (iv) uncontrolled diabetes mellitus (defined as HbA1c ≥ 9% or 75 mmol/L or with chronic complications), (v) any immunodeficiency condition (both primary or secondary), (vi) chronic obstructive pulmonary disease and/or other chronic respiratory disease in subject ≥ 55 years; number of medical condition which qualified the patient for mAb therapy (one versus ≥ 2); SARS-CoV-2 vaccination status (defined as the receipt of at least one dose of SARS-COV-2 vaccination ≥ 14 days before the confirmed diagnosis of SARS-CoV-2 infection); and type of mAb regimen.

### Outcomes

The primary outcome was hospitalisation for any cause within 28 days after mAb infusion. Secondary outcomes were (i) COVID-19-related hospitalisation, defined as the diagnosis of SARS-CoV-2 pneumonia requiring oxygen replacement therapy, (ii) use of mechanical ventilation and (iii) death within 28 days of mAb infusion. Outcome variables were collected within 28 days by means of a follow-up evaluation by phone or by reviewing clinical charts or AIFA web-data repository, on the basis of the feasibility of each hospital.

### Virological and serological analyses

Only for those patients who signed an additional informed consent, an NPS and a serum sample at the time of mAb infusion (baseline) were collected for SARS-CoV-2 variant identification and anti-SARS-CoV-2 antibody quantification. RNA was extracted using the MagMAX Viral Pathogen II Nucleic acid kit (Thermo Fisher) on a KingFisher Flex Purification System according to the manufacturer’s instructions. Extracted RNA was subjected to Real-Time (RT) Reverse Transcriptase (RT-)qPCR using the TaqPath™ COVID-19 CE-IVD RT-PCR Kit (Thermo Fisher) on a QuantStudio™ 5 RT-PCR instrument (384-well block, 5 colours, Thermo Fisher) which detects three genes in the SARS-CoV-2-viral genome: S protein, N protein and ORF1ab genes. In case of positive RT-qPCR (detection of the MS2 phage–positive control and at least two gene targets), the extracted RNA was subjected to automated cDNA conversion and multiplexed library preparation using the Illumina COVIDSeq Test kit (Illumina Inc.) on a Zephyr G3 NGS (PerkinElmer) instrument according to the manufacturer’s instruction. Pooled libraries were sequenced using the High Output Kit v2 (Illumina Inc.) with a 1.4-nM PhiX Library positive control v3 using a 1% spike-in on a NextSeq 500/550 instrument (Illumina Inc.). Raw sequencing data quality for each sample was assessed using FastQC (https://www.bioinformatics.babraham.ac.uk/projects/fastqc/) followed by quality trimming using a Phred score cut-off of 25 with TrimGalore v. 0.6.7 (https://github.com/FelixKrueger/TrimGalore). Sequencing was considered successful if an estimated genome coverage > 100 × was obtained. Read mapping was performed against the SARS-CoV-2 genome (GenBank: NC_045512.2) using the CLC Genomics Workbench v.9.5.3 (Qiagen) with a length and a similarity fraction of 0.5 and 0.8, respectively. Consensus sequences were extracted, and lineages assigned using Phylogenic Assignment of named Global Outbreak LINeages (PANGOLIN) [9]. Patients’ antibody responses were characterised by quantitative assessment of anti-spike, anti-receptor-binding domain (RBD) and anti-nucleocapsid (NC) IgG. Quantitative IgG results were measured in antibody units (AU) per millilitre and converted to WHO binding antibody units (BAU) per millilitre [10].

### Statistical analysis

Descriptive analyses were computed on the total population and by type of mAb regimen. Baseline demographic and clinical variables as well as outcome variables of subjects receiving the three different mAb regimens were assessed and compared using chi-square ‘χ^2^’ for categorical variables and Kruskal–Wallis test for continuous variables. Categorical and continuous variables were expressed as median and Q1–Q3 and frequency and proportions, respectively. In the outcome assessment, patients who were treated with mAb but admitted to hospital 24 h or less after the infusion time were excluded, since these events were likely related to a rapid progression of illness per se, regardless of the mAb infusion. Factors associated with the primary outcome variable were identified using a two-level multilevel multivariate logistic regression (MMLR) model with a random intercept at the hospital level. The random component accounted for the hierarchical nature of the data collected across hospitals. The likelihood ratio (LR) test was used to compare ordinal multivariable logistic regression (OMLR) with MMLR. The final model estimated the adjusted odds ratios (aORs) along with 95% confidence interval (95% CI) for factors associated with 28-day all-cause hospitalisation. Wald tests were used for testing the difference between variable levels in the model. A *p* value less than 0.05 was regarded as statistically significant. All analyses were conducted with STATA®, version 17.0 (College Station, TX: StataCorp LP).

## Results

### Description of the study population

From 18 March to 15 June, a total of 1132 patients were screened using the electronic reporting system. Among them, 653 mild-to-moderate COVID-19 outpatients were included in the study and treated with mAb. After the exclusion of 18 patients who were admitted to the hospital within 24 h from mAb infusion, a total of 635 patients were included in the analysis with a 28-day follow-up. Most patients were male (391, 61.6%) with a median age of 64 years (Q1–Q3, 56–73). The most common conditions which qualified patients to mAb therapy were cardio-cerebrovascular disease (360 patients, 56.7%) and BMI ≥ 35 m^2^/kg (183, patients, 28.8%). The cohort included 188 patients (29.6%) with at least two medical conditions. The median time from symptom onset to mAb administration was 5 days (Q1–Q3, 4–7); 98 out of 636 patients (15.4%) received mAb therapy within 48 h from symptom onset. At the time of study inclusion, 15 patients (2.4%) and 80 patients (12.6%) had received full and partial SARS-CoV-2 vaccination courses, respectively. Patients’ characteristics are shown in Table 2.

**Table 2:** Baseline demographic and clinical data on 635 patients receiving mAb therapy for COVID-19 by mAb regimen

Variables	All patients (*N* = 635)	Bamlanivimab (*N* = 161)	Bamlanivimab-etesevimab (*N* = 396)	Casirivimab-imdevimab (*N* = 78)	*p* value
Demographic characteristics					
Age, years, median (Q1–Q3)	64 (56–73)	63 (56–73)	64 (57–74)	62 (53–70)	0.15
Age group, *n* (%)					
< 65 years	327 (51.5)	82 (50.9)	200 (50.5)	45 (57.7)	0.50
≥ 65 years	308 (48.5)	73 (49.1)	196 (49.5)	33 (42.3)	
Gender, *n* (%)	391 (61.6)	110 (68.3)	234 (59.1)	47 (60.3)	0.12
Male					
Body mass index, median (Q1–Q3)	29 (26–36)	29 (25–36)	30 (25–36)	29 (26–37)	0.67
Clinical characteristics					
Condition qualifying for mAb therapy, *n* (%)					
Body mass index ≥ 35 kg/m^2^	183 (28.8)	49 (30.4)	113 (28.5)	21 (26.9)	0.84
Chronic dialysis	11 (1.7)	5 (3.1)	5 (1.3)	1 (1.3)	0.29
Cardio-cerebrovascular disease ≥ 55 years	360 (56.7)	88 (54.7)	236 (59.6)	36 (46.1)	0.08
Uncontrolled or complicated diabetes mellitus	105 (16.5)	20 (12.4)	74 (18.7)	11 (14.1)	0.17
Any immunodeficiency condition	92 (14.5)	25 (15.5)	55 (13.9)	12 (15.4)	0.83
Chronic respiratory disease ≥ 55 years	107 (16.9)	28 (17.4)	63 (15.9)	16 (20.5)	0.57
1 comorbidity	447 (70.4)	117 (72.7)	270 (68.2)	60 (76.9)	0.24
≥ 2 comorbidities	188 (29.6)	44 (27.3)	126 (31.8)	18 (23.1)	
Time from symptoms onset to infusion, days (median, Q1–Q3)	5 (4–7)	5 (4–7)	6 (4–7)	5 (4–7)	0.30
Vaccination status					
SARS-CoV-2 vaccination, *n* (%)	95 (15.0)	11 (6.8)	63 (16.1)	20 (27.0)	< 0.001
Completed course	15 (2.4)	1 (0.6)	6 (1.5)	8 (10.3)	
Partial course	80 (12.6)	10 (6.2)	57 (14.4)	12 (16.7)	

### Virological and serological results

Laboratory analyses were conducted in a subgroup of 169 patients. Sequencing was successfully performed in 149 (88%) patients. Among them, 147 patients were infected by the B.1.1.7 (Alpha, UK) variant, while two patients were infected by B.11.462. Both are sub-lineages of the B.1 European clade, but not directly related with each other. Anti-SARS-CoV-2 IgGs were successfully quantified in 169 patients: 44 (26%), 108 (64%) and 17 (10%) received bamlanivimab, bamlanivimab-etesevimab and casirivimab-imdevimab, respectively (Appendix Table 5). No significant differences were observed among the three different arms of treatment regarding the vaccination status (*p* = 0.644). Anti-spike, RBD or NC antibodies across the three mAb regimen groups showed no significantly different titres. Anti-NC titres remained low for all three groups, whereas anti-spike and anti-RBD titres exceeding mid-immunoglobulin levels defined by WHO International Standards for anti-SARS-CoV-2 immunoglobulins were shown in four patients (Appendix Figure 2).

### Characteristics of the three mAb study group

During the study period, the most prescribed mAb regimen was bamlanivimab-etesevimab (396 patients, 62.4%), followed by bamlanivimab (161 patients, 25.4%) and casirivimab-imdevimab (78 patients, 12.2%) (Appendix Figure 3). The three mAb groups were similar in terms of age, gender, comorbidities distribution and time of treatment (Table 2). The percentage of patients who had received a partial or complete SARS-CoV-2 vaccination course at the time of study inclusion were significantly higher (*p* < 0.001) in the casirivimab-imdevimab group (27.0%) compared to the bamlanivimab (6.8%) and bamlanivimab-etesevimab (16.1%) groups. The difference was due to time discrepancy between the vaccination campaign, which started in Italy on 27 December 2020 and the distribution of casirivimab-imdevimab, which was made available in late March 2021.

### Clinical outcomes by mAb regimen

The overall 28-day all-cause hospitalisation rate was 11.3% (72/635 patients). Stratifying by mAb regimen, the hospitalization rate resulted significantly higher (*p* 0.001) in patients treated with bamlanivimab (30 patients, 18.6%) compared to patients who received bamlanivimab-etesevimab (40 patients, 10.1%) and casirivimab-imdevimab (2 patients, 2.6%) (Table 3). The median time from microbiological diagnosis to hospitalisation was 7 days (Q1–Q3, 4–10) and similar across the three mAb cohorts. Most 28-day hospitalisations were due to COVID-19 (53 patients, 8.6%): 23 patients (14.9%) in the bamlanivimab group, 28 patients (7.3%) in the bamlanivimab-etesevimab group and 2 patients (2.6%) in the casirivimab-imdevimab group (*p* 0.003). Fourteen out of 53 (26.4%) hospitalised patients required mechanical ventilation (non-invasive ventilation: 11 patients; orotracheal intubation: 3 patients). Table 3 shows the clinical outcomes.

**Table 3:** Primary and secondary outcomes by total population and by mAb regimen

Outcome	All patients (*N* = 635)	Bamlanivimab (*N* = 161)	Bamlanivimab-etesevimab (*N* = 396)	Casirivimab-imdevimab (*N* = 78)	*p* value
28-day all cause hospitalisation, ***n*** (%)	72 (11.3)	30 (18.6)	40 (10.1)	2 (2.6)	0.001
COVID-19-related hospitalisation	53 (8.6)	23 (14.9)	28 (7.3)	2 (2.6)	0.003
Need of mechanical ventilation	14 (2.2)	8 (4.9)	5 (1.2)	1 (1.2)	0.814
Death	6 (0.9)	3 (1.9)	3 (0.7)	0 (0.0)	0.31

Overall, six deaths occurred: three patients in the bamlanivimab group and three patients in the bamlanivimab-etesevimab group. All patients suffered from at least two comorbidities. The cause of death was respiratory failure due to COVID-19 pneumonia. None of the patients had received SARS-CoV-2 vaccination.

### Factors associated with 28-day all-cause hospitalisation

Based on the bivariate analysis, patients who received bamlanivimab-etesevimab (OR 0.52, 95% CI: 0.31–0.87, *p* 0.014) and casirivimab-imdevimab (OR 0.12, 95% CI 0.03–0.54, *p* 0.006) were less likely to experience 28-day all-cause hospitalisation compared with patients who received bamlanivimab. Other variables which resulted significantly associated with the outcome were age (OR 1.03, 95% CI 1.01–1.05. *p* 0.003) and male gender (OR 2.01, 95% CI 1.14–3.54. *p* 0.016) (Table 4). As for the SARS-CoV-2 vaccination, no patients experienced the primary outcome and therefore this variable was not included in the model as it predicted perfectly the outcome. After adjustment for significant variables, MMLR showed that both bamlanivimab-etesevimab (aOR 0.51, 95% CI 0.30–0.88, *p* 0.015) and casirivimab-imdevimab (aOR 0.14, 95% CI 0.03–0.61, *p* 0.009) remained significantly associated with decreased odds of 28-day all-cause hospitalisation, compared to bamlanivimab. Age (aOR 1.03, 95% CI 1.01–1,05 *p* 0.012) and gender (male, aOR 2.10, 95% CI 1.15–3.82, *p* 0.015) were confirmed as independent variables related to the outcome. The LR test (*χ*^2^ = 3.68, *p* ≤ 0.028) resulted significant, suggesting the need of accounting for hospital variability with a random intercept (Table 4).

**Table 4:** Factors related to 28-day all-cause hospitalisation on the two-level multilevel logistic regression model

All-cause 28-day hospitalisation				
Variable	Bivariate		Multivariate	
	OR (95% CI)	***p-***value	aOR (95% CI)	***p-***value
mAb regimen (reference: bamlanivimab)				
Bamlanivimab-etesevimab	0.52 (0.31–0.87)	0.014	0.51 (0.30–0.88)	0.015
Casirivimab-imdevimab	0.12 (0.03–0.54)	0.006	0.14 (0.03–0.61)	0.009
Baseline characteristics				
Age	1.03 (1.01–1.05)	0.003	1.03 (1.01–1.05)	0.012
Gender	2.01 (1.14–3.54)	0.016	2.10 (1.15–3.82)	0.015
BMI > 35 kg/m^2^	0.63 (0.35–1.15)	0.136		
Number of comorbidities	1.06 (0.61–1.82)	0.827		
Uncontrolled or complicated diabetes	0.88 (0.44–1.77)	0.727		
Any immunodeficiency condition	1.49 (0.76–2.89)	0.244		
Cardio- cerebrovascular disease ≥ 55 years	1.18 (0.68–1.99)	0.537		
Chronic respiratory disease ≥ 55 years	1.44 (0.78–2.66)	0.236		
Chronic dialysis	0.65 (0.08–5.27)	0.689		
Time from symptoms onset to mAb infusion	0.99 (0.89–1.13)	0.915		
Constant			0.02 (0.01–0.10)	
Center ơ^2^			0.25 (0.04– 1.50)	

LR test versus logistic regression: Chibar2 = 3.68 Prob ≥ Chibar2 0.028

**Fig. 1:**
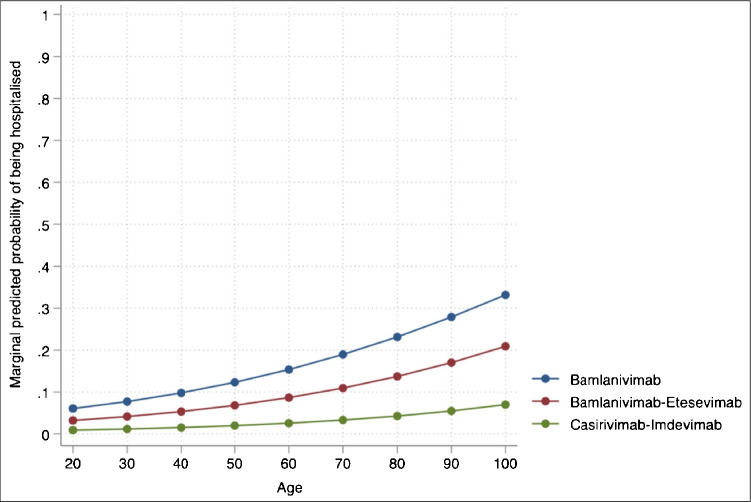
Plots of marginal predicted probabilities of 28-day all-cause hospitalisation by age and mAb regimen

Based on the MMLR, marginal predicted probabilities of being hospitalised within 28 days after mAb infusion were estimated by mAb regimen and age, as graphically displayed in Fig. 1. The probability of experiencing the outcome variable increases independently with the increasing of age. The curves of the three mAb regimens show a lower probability of being hospitalised among patients of younger age while; with the age increase, the difference between regimens becomes more substantial. As estimated by the model and confirmed by the respective ORs, the outcome variable is significantly influenced by the mAb regimens, showing for both casirivimab-imdevimab (aOR 0.14) and bamlanivimab-etesevimab (aOR 0.51) a lower probability of hospitalisation compared to bamlanivimab alone. The difference between casirivimab-imdevimab and bamlanivimab-etesevimab resulted statistically not significant (Wald test, *χ*^2^ = 2.46, *p* 0.119).

## Discussion

To our knowledge, our study is the first to report data on comparative efficacy of three different mAb regimens in a prospective cohort of mild-to-moderate COVID-19 adult outpatients at high risk for disease progression. The overall hospitalisation rate was 11.3%. Stratifying by mAb regimen, the rate increased up to 18.6% in the bamlanivimab group versus 10.1% in the bamlanivimab-etesevimab group and 2.6% in the casirivimab-imdevimab groups. Data from the Italian national COVID-19 surveillance program in the same study period shows a hospitalisation rate of 25% in patients aged > 65 years regardless of the presence of comorbidity [11]. In placebo-controlled RCTs, the hospitalisation rates in patients receiving bamlanivimab, bamlanivimab-etesevimab and casirivimab-imdevimab were 1.6%, 2.1% and 3%, respectively [5–7]. A possible reason explaining this outcome discrepancy lies in the different selections of the study population. Compared to these RCTs, our cohort included patients at a higher baseline risk for poor outcome, according to the a priori definition by AIFA EUA and about one-third of patients suffered from at least two medical conditions. The evidence from RCT on the use of mAb in populations with high comorbidity burden is very limited. The BLAZE-1 ad interim post hoc subgroup analysis performed on 215 patients at high risk of disease progression (BMI ≥ 35 kg/m^2^ or ≥ 65 years) reported a 4.2% hospitalization rate [12]. Case–control studies have demonstrated a hospitalisation risk reduction of 50% in subjects treated with either bamlanivimab or casirivimab-imdevimab and 64% in subjects treated with bamlanivimab, compared to historical and contemporary untreated controls, respectively [13, 14]. The only study providing comparative efficacy between mAb regimens, performed by Mayo Clinic on 3594 high-risk outpatients receiving bamlanivimab monotherapy and casirivimab-imdevimab, found a difference of 1.5% between the two mAb in the composite hospitalisation/ED visit outcome [15] .

In our study, all patients with a partial or full SARS-CoV-2 vaccination course did not experience progression of COVID-19, irrespective of the mAb regimen administered, confirming that the currently authorised SARS-CoV-2 vaccines show 85 to 96% of efficacy in preventing moderate to severe COVID-19 [16]. Worthy of note, the 15 fully vaccinated patients with breakthrough infection had all severe comorbidities and half of them suffered from an immunocompromised condition which may have affected a proper antibody response to vaccine. A large Center for Disease Control and Prevention breakthrough infection surveillance on about 10,000 subjects revealed that up to 30 April in USA, 10% of patients developing breakthrough infections were hospitalized [17]  So far, no studies have been published on the use of mAb in outpatients with breakthrough infections.

The SARS-CoV-2 genome analysis obtained from NPS revealed that 99% of the typeable viral strains circulated in the area were classified as B.1.1.7/alpha lineage, against which bamlanivimab retained in vitro activity [18]. Although the variant determination was not conducted to the whole population, epidemiological data confirmed that the alpha strain was dominant in the same geographical region in the study period [19]. According to this finding, no difference in activity among the three mAb regimens was expected.

This study has some limitations. First, no random allocation of mAb regimen was adopted for homogenising the number and baseline characteristics of patients in each mAb group; this drawback was partly mitigated by the fact that AIFA EUA criteria uniformed the patients into a high-risk category and an MMLR approach was used to optimize the control of confounders. Second, the uniform baseline serology seems to suggest that vaccination status did not contribute to the observed differences in the clinical outcomes for the three mAb regimen groups. Moreover, it is recognized that T-cell response may contribute to clinical protection; unfortunately, no data about cellular immunity were available. Third, the study was conducted in a regional setting in Italy and the external validity to other health care systems or geographical areas with different epidemiology patterns is unknown. Fourth, about two-thirds of patients received mAb between the 5th and 7th days of symptom onset and this delay, due to real-life usage, may have compromised the treatment efficacy.

With limited therapeutic armamentarium for COVID-19 outpatients and decreased efficacy of vaccination in the fragile population [20], bamlanivimab-etesevimab and casirivimab-imdevimab can provide a viable option for high-risk patients for disease progression when administered early in the course of disease, avoiding hospitalisation and mitigating the healthcare burden. So far, no firm conclusions on efficacy of the various mAb regimens can be drawn from currently published RCTs [21], especially in high-risk populations. In the light of the rapid emergence and spread of viral variants with potential impact on vaccine-induced immunity, mAb therapy may become a key strategy also for the treatment of breakthrough infections. Viral variants may also impair the activity of mAb themselves; therefore, the establishment of local genomic surveillance is crucial to guide the selection of mAb at patient level. Future research should focus on the use of mAb in the fragile, vaccinated population and in patients with breakthrough infections in order to understand if the difference in efficacy is confirmed in RCTs. These data will be helpful in driving the design of such studies. Clinical decision-making algorithms combining patient-risk profile, SARS-CoV-2 vaccination status and epidemiological data (local surveillance of circulating variants) are needed to tailor mAb therapy and optimise resource allocation.

## Figures and Tables

**Fig. 2:**
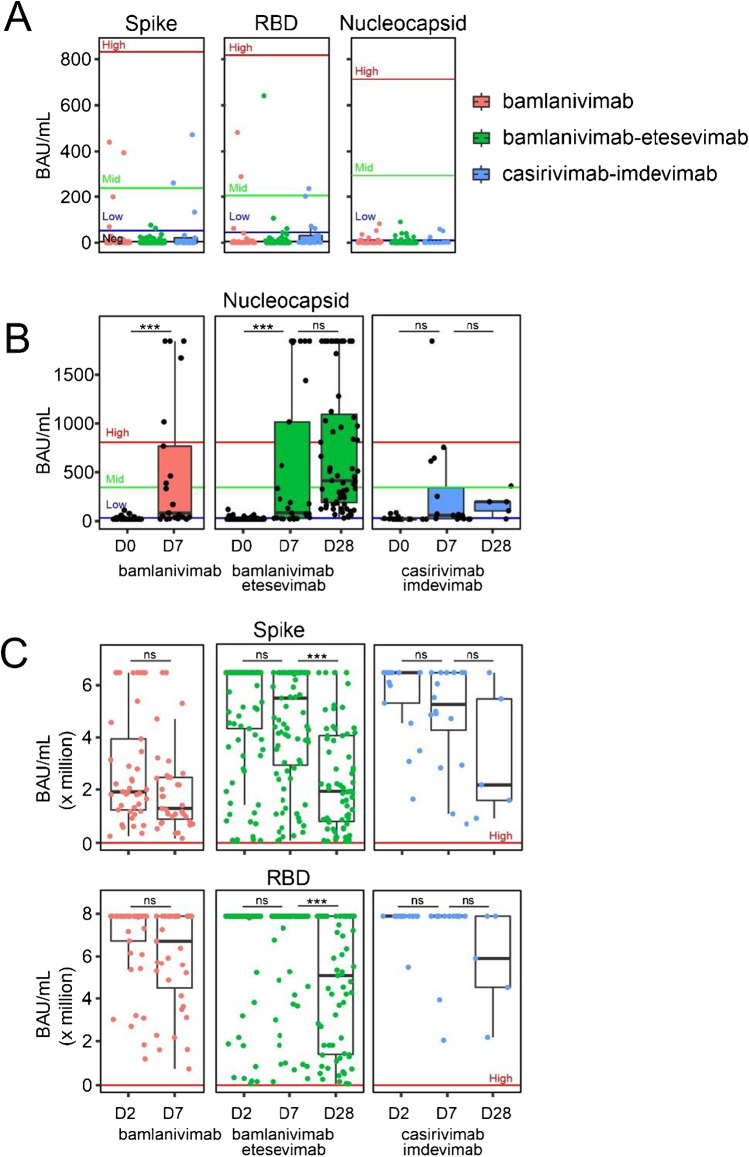
Serology in a sub-selection of patients receiving mAb therapy. **a** Anti-nucleocapsid, anti-RBD and anti-spike measurement in patients prior to the administration of mAb therapy to study baseline anti-SARS-CoV-2 immunity. Serum samples from the bamlanivimab (*n* = 44), bamlanivimab/etesevimab (*n* = 108) and casirivimab/imdevimab (*n* = 17) collected prior to mAb administration were measured at 1:1000 dilution. Low, mid and high lines indicate the SARS-CoV-2 WHO standards. **b** Natural immunity assessment after day 7 and day 28 of mAb treatment. Anti-nucleocapsid measurements at day 7 (bamlanivimab *n* = 21; bamlanivimab-etesevimab, *n* = 29; and casirivimab-imdevimab, *n* = 16) and day 28 (bamlanivimab/etesevimab, *n* = 67; casirivimab/imdevimab, *n* = 5) were compared with anti-nucleocapsid titres at the timepoint before infusion. **c** Synthetic mAb stability studied with anti-spike and anti-RBD measurements at day 7 and day 28 after mAb treatment on samples enumerated in **b*.*** Box plots indicate median (middle line), 25th and 75th percentiles (box) and 5th and 95th percentiles (whiskers) as well as outliers. ***p < 0.001. **p < 0.010. *p < 0.050. ns: non-significant

**Fig. 3:**
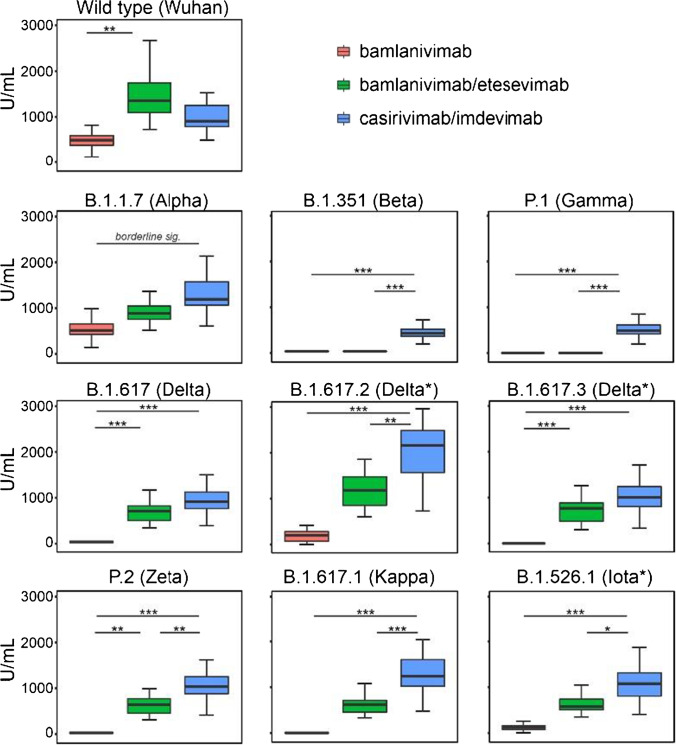
Seroneutralization in a sub-selection of patients receiving mAb therapy. Anti-RBD, Ant-Spike (WT, Wuhan) and Anti-Spike (B.1.1.7/alpha) measurements in patients treated with bamlanivimab (*n* = 41 (D2) and *n* = 35 (T2), bamlanivimab-etesevimab (*n* = 97 (D2), *n* = 91 (D7), *n* = 67 (D28)) and casirivimab-imdevimab (*n* = 17 (D2), *n* = 16 (D7), *n* = 5 (D28). (D) Neutralizing antibody measurements against 10 different SARS-CoV-2 variants of concern (VOCs). Box plots indicate median (middle line), 25th and 75th percentiles (box), and 5th and 95th percentiles (whiskers). ***p < 0.001. **p < 0.010. *p < 0.050. ns: non-significant

## Data Availability

The datasets generated during and/or analysed during the current study are available from the corresponding author on reasonable request.

## Appendix

**Table 5:** Kinetics of monoclonal antibodies (mAbs). D2: 2 ± 1 days after mAb infusion. D7: 7 ± 2 days after mAb infusion. D28: 28 days after mAb infusion. CI: confidence interval

mAb	Timepoint	Spike (BAU/mL)		RBD (BAU/mL)	
		Average (95% CI)	Lowest	Average (95% CI)	Lowest
Bamlanivimab	D2	2,786,835 (2,133,560–3,440,111)	204,440	6,252,371 (5,448,762–7,055,979)	1,170,685
	D7	1,928,734 (1,388,358–2,469,110)	123,730	5,928,446 (5,163,756–6,693,135)	700,486
	D28	-	-	-	-
Bamlanivimab/etesevimab	D2	5,142,686 (4,720,248–5,565,124)	38,723	5,731,598 (5,055,672–6,407,523)	107,087
	D7	4,534,502 (4,082,631–4,986,373)	36,162	6,119,030 (5,501,906–6,736,154)	100,927
	D28	2,409,495 (1,933,923–2,885,066)	6,902	4,500,000 (3,774,716–5,225,284)	6,933
Casirivimab/imdevimab	D2	5,590,384 (4,876,624–6,304,144)	1,631,223	6,353,212 (4,885,599–7,820,825)	5,460,613
	D7	4,844,247 (3,884,300–5,804,194)	674,784	6,782,726 (5,566,251–7,999,201)	2,042,686
	D28	3,324,023 (1,136,875–5,511,170)	893,507	5,676,534 (3,556,361–7,796,708)	2,180,823
